# Pyroptosis: Role and Mechanisms in Cardiovascular Disease

**DOI:** 10.3389/fcvm.2022.897815

**Published:** 2022-05-11

**Authors:** Xinzhe Chen, Peng-Chao Tian, Kai Wang, Man Wang, Kun Wang

**Affiliations:** ^1^Institute of Translational Medicine, The Affiliated Hospital of Qingdao University, College of Medicine, Qingdao University, Qingdao, China; ^2^State Key Laboratory of Cardiovascular Disease, Heart Failure Center, National Center for Cardiovascular Diseases, Peking Union Medical College, Fuwai Hospital, Chinese Academy of Medical Sciences, Beijing, China

**Keywords:** pyroptosis, gasdermin, inflammasome, caspase, cardiovascular disease

## Abstract

Cardiovascular disease (CVD) is a common disease that poses a huge threat to human health. Irreversible cardiac damage due to cardiomyocyte death and lack of regenerative capacity under stressful conditions, ultimately leading to impaired cardiac function, is the leading cause of death worldwide. The regulation of cardiomyocyte death plays a crucial role in CVD. Previous studies have shown that the modes of cardiomyocyte death include apoptosis and necrosis. However, another new form of death, pyroptosis, plays an important role in CVD pathogenesis. Pyroptosis induces the amplification of inflammatory response, increases myocardial infarct size, and accelerates the occurrence of cardiovascular disease, and the control of cardiomyocyte pyroptosis holds great promise for the treatment of cardiovascular disease. In this paper, we summarized the characteristics, occurrence and regulation mechanism of pyroptosis are reviewed, and also discussed its role and mechanisms in CVD, such as atherosclerosis (AS), myocardial infarction (MI), arrhythmia and cardiac hypertrophy.

## Introduction

Cell death is a complex biological process that regulates various physiological functions and maintains homeostasis. On the one hand, cell death ensures normal host development by clearing out old, damaged and useless cells, for another, abnormal cell death destroys organ functions and causes inflammation when cells cannot maintain essential life functions. Cell death is classified into two categories based on functional differences: accidental cell death (ACD) and regulated cell death (RCD), RCD also known as programmed cell death (PCD). Currently, known types of RCD include apoptosis, necrosis, pyroptosis, ferroptosis, alkali death, etc. (1–4), this review focuses on the molecular mechanisms of pyroptosis. Rupture of the plasma membrane (PMR) during pyroptosis is an irretrievably catastrophic event. PMR rupture releases intracellular molecules called damaged associated molecular patterns (DAMPs) during pyroptosis, which promotes an inflammatory response (5). Pyroptosis is a caspase-dependent form of cell death found in immune cells during microbial infection (6). In 1992, Zychlinsky et al.’s found that Shigella could cause macrophage death. Electron microscopy found that chromatin condensation, membrane blebbing, and DNA fragmentation were identified as apoptosis (7). By 1994, the team discovered that Shigella can release large amounts of IL-1 after infecting macrophages (8), and believed that this form of death was accompanied by an inflammatory response. In 1996, Chen et al. reported the activation of cysteine-containing aspartate proteolytic enzyme (caspase-1) in this cell death, which was the first report that caspase-1 could cause cell death (9). Until 2001, the Boise team discovered the pro-inflammatory cell death method caused by caspase-1, and first proposed the concept of cell pyroptosis (6). Pyroptosis is a kind of programmed cell death, but different from other programmed death, the main caspase executing death is caspase -1/4/5/11 (10). Pyroptosis can also mediate IL-1β, IL-18 and GSDMD cleavage (10, 11), caspase-mediated cleavage of a member of the GSDM family is a necessary step to trigger cell pyroptosis (12). In addition to the release of two specific pro-inflammatory cytokines, IL-1β, and IL-18, other inflammatory mediators include DNA fragments, high mobility group protein B1, adenosine triphosphate, and lipid mediators (13, 14). However, activation of inflammasome and GSDMD does not always trigger significant cell lysis (15–17). A recent study reported that pyroptosis captures live bacteria (18), the presence of cytoskeletal structures that capture bacteria until dead cells are engulfed by phagocytes (19). In conclusion, on the one hand, pyroptosis triggers the occurrence, and development of cardiovascular diseases by releasing inflammatory factors and amplifying the inflammatory response cascade, and on the other hand, it can trap bacteria through the cytoskeleton and trigger the host immune response.

## The Pyroptosis Pathway

**FIGURE 1:**
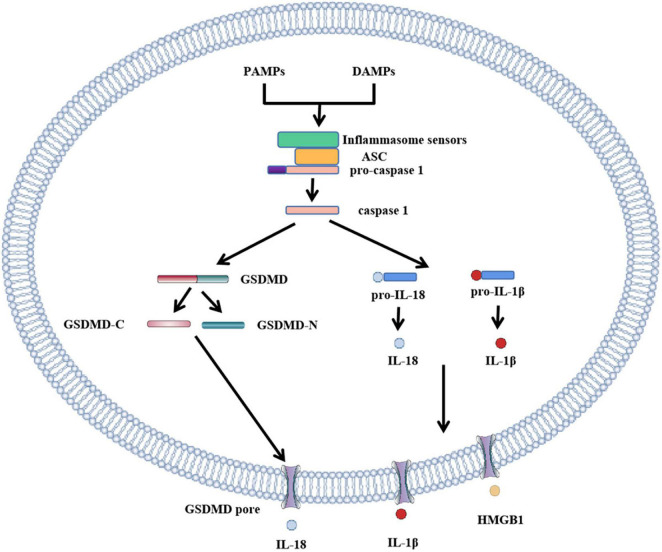
Role of GSDMD in canonical inflammasome activation. The canonical pathway of pyroptosis. PAMPs or DAMPs bind to inflammasome to activate caspase-1, cleaving GSDMD to form GSDMD-C and GSDMD-N. GSDMD-N cluster and bind to the plasma membrane to form GSDMD pores. Simultaneously activated caspase-1 activates IL-18 and IL-1β, and cytokines IL-18 and IL-1β are released into the cell through the GSDMD pore. In addition to IL-18 and IL-1β, HMGB1 is also removed.

**FIGURE 2:**
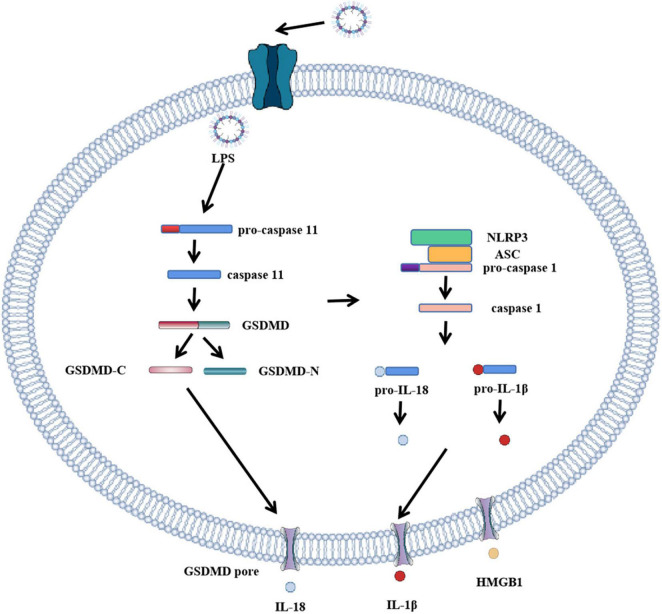
Role of GSDMD in non-canonical inflammasome activation. The canonical pathway of pyroptosis. When LPS enters cells, it directly participates in and activates human caspase-4 and caspase-5 and mouse caspase-11. Caspase-11 cleaves GSDMD, forming GSDMD-C and GSDMD-N. GSDMD-N cluster and bind to the plasma membrane. Formation of GSDMD pore, the formation of GSDMD pore activates inflammasome to bind to caspase-1, activated caspase-1 activates IL-18 and IL-1β, and cytokines IL-18 and IL-1β are released extracellularly through GSDMD pore.

Pyroptosis pathways include classical pathways and non-classical pathways. In addition, studies have shown that apoptosis-related caspase-3 and caspase-8 also trigger pyroptosis. In the classical pathway, pro-IL-1β and pro-IL-18 are cleaved by caspase-1 to form activated IL-1β and IL-18, full-length GSDMD was also cleaved by caspase-1 to N-terminal and C-terminal GSDMD during pyroptosis. The GSDMD-N-terminal binds to the plasma membrane and rapidly forms pores 12–14 nm in diameter, leading to cytoplasmic swelling. The plasma membrane lyses and releases pro-inflammatory cell contents. In addition to caspase-1, the researchers found a similar phenomenon in caspase-4, caspase-5, and caspase-11, which also cause pyroptosis. When bacterial lipopolysaccharide (LPS) enters the cytoplasm, it directly activates caspase-4, caspase-5 in humans and caspase-11 in mice (10, 20) and subsequently causes GSDMD cleavage. The pyroptosis pathway independent of caspase-1 is called the non-classical cell pyroptosis pathway (21, 22), GSDMD pore triggers assembly of NLRP3 inflammasome and maturation of caspase-1 and then activates caspase-1 which causes the cleavage of IL-1β and IL-18. Recent studies have shown that OspC3 in shigella III type secretory system blocks activation of caspase-4 or caspase-11, and GSDMD cleavage, thereby preventing the occurrence of pyroptosis and evading the host immune system (23). The pyroptosis pathway is shown in Figure 1 and Figure 2: canonical and noncanonical pathways.

## Key Components Involved in Pyroptosis

Gasdermin, caspase and inflammasomes are important molecules in pyroptosis. Their characteristics and function in pyroptosis will be discussed in the following sections.

## The Gasdermin Family

In humans, the GSDM family is made up of six members: GSDMA, GSDMB, GSDMC, GSDMD, GSDME, and GSDMF, as shown in Table 1. Mice lacked GSDMB but expressed GSDMA and GSDMC (24). In terms of structure, except for GSDMF, all GSDM family members have three common structural domains, one N-terminal hole formation domain, one C-terminal self-inhibition domain, one binding domain, in which the N-terminal and C-terminal form an inactive full-length gasdermin protein through the binding domain. As the executor of pyroptosis, GSDM is associated with many diseases. The key role of GSDMD in pyroptosis is due to its high expression in a variety of tissues. GSDMD induces pyroptosis and further amplifies inflammatory response with intracellular material outflow, which is connected with the nosogenesis of many inflammatory autoimmune diseases (25, 26). In addition, spontaneous mutations in GSDMs cause hair loss (GSDMA) (27), asthma (GSDMB) (28), hearing impairment (GSDME/DFNB59) (29). There is increasing evidence that GSDMs perform different functions under different caspase activation, including tumor pyroptosis and pathogen infection (30, 31). Because DNA methyltransferase inhibitors induce GSDMs expression, some GSDMs have potential applications in epigenetics modification, especially DNA methylation, of which DNA methylation of GSDMA and GSDME is the most obvious (32, 33).

**TABLE 1:** Characteristics of GSDM family proteins.

Gene	Activate way	Organism	Disease links	References
GSDMA	Autoactive	Human and mouse	Alopecia	(71)
GSDMB	GzmA Caspase-4	Human	Autoimmune disease, breast cancer, asthma, uterine cervix cancer, tumor	(43, 45, 46, 72)
GSDMC	Caspase-8	Human and mouse	Tumor, colorectal cancer	(49, 51)
GSDMD	Caspase-1/4/5/11	Human and mouse	Autoimmune encephalomyelitis, Yersinia infection, familial Mediterranean	(25, 73, 74)
GSDME	Autoactive Caspase-3 GzmB	Human and mouse	Tumor, hearing impairment	(29, 53, 66)
GSDMF	Not known	Human and mouse	Auditory neuropathy	(75)

### GSDMs Cause Hair Loss

The first member of the GSDM family, GSDMA, was identified by mapping genes that cause abnormal skin and hair development in cloned mice Rim3 mutants (34). There is increasing evidence that GSDMA is closely linked to skin diseases. TNF can upregulate GSDMA3 and cause apoptosis in mouse skin keratinocytes (35). GSDMA has also been associated with susceptibility to asthma, and macrophage transcriptome analysis has revealed that GSDMA is associated with the pathogenesis of sclerosis and inflammatory bowel disease (36–38).

### GSDMB

GSDMB is more widely expressed than GSMDA, especially in the gastrointestinal epithelium, liver, neuroendocrine and immune cells (39–41). There are four subtypes of GSDMB, and caspase-4 promotes pyroptosis by activating GSDMB. In another large-scale genome-wide analysis of asthma, mutations in the GSDMB gene were associated with childhood asthma and autoimmune diseases (42–44). In addition, the expression of GSDMB in tumor cells increased and promoted the growth, invasion, metastasis of neoplasm (45–47).

### GSDMC

GSDMC was originally discovered in melanoma cells. With the further study, researchers found that the expression of GSDMC in melanoma cells has been at a high level (48). Researchers defined GSDMC as a marker of melanoma occurrence, and GSDMC is expressed in a number of cells and tissues, and is also one of the star molecules. The deactivation of transforming growth factor β receptor Type II leads to the up-regulation of GSDMC and promotes the proliferation of colorectal cancer (49). Studies have shown that the Death receptor 6 can recruit pro-caspase-8 and GSDMC, activate caspase-8 to cleave GSDMC and thus induce pyroptosis (50). A recent study has shown that hypoxia triggers the binding of signal transducer and activator of transcription(STAT3)phosphorylation and programmed death ligand 1(PD-L1)in tumor cells, thereby causing nuclear translocation of PD-L1 to induce GSDMC expression. Caspase-8 specifically cleaves GSDMC and induces cell pyroptosis. PD-L1 mediates GSDMC expression under the activation of tumor necrosis factor α(TNF-α). The apoptosis of cancer cells is transformed into pyroptosis, which promotes tumor death (51).

### GSDMD

GSDMD is the most studied protein in pyroptosis, which is mainly expressed in immune cells, placenta, gastrointestinal epithelial cells, various cancers, and Jurkat T and Ramos B cancer cell lines (52). It is a crucial executor of pyroptosis, mediating inflammatory responses and influencing chronic inflammatory diseases. When the inflammasome complex is formed, GSDMD can recruit caspase, which cleaves Asp275 (mouse Asp276) of GSDMD in the linker (53), releasing the self-inhibition of its GSDMD-N domain and performing pyroptosis through pore-forming. GSDMD-induce pyroptosis of macrophages also activate the release of cytokines that activate coagulation and lead to sepsis (54, 55). Similarly, GSDMD relies on neutrophils to capture platelets and promotes thrombosis and helps to activate clotting (56). Studies of gene defects or gene knockdown have also shown that GSDMD is associated with the nosogenesis of alcoholic hepatitis, non-alcoholic steatohepatitis, non-infectious liver injury, and ischemia/reperfusion (I/R) injury (57–60).

### GSDME

GSDME, also known as DFNA5, was initially being identified as a gene associated with deafness (61), mutations of DFNA5 lead to hearing impairment, and mutations in several different DFNA5 leads to a jump exon eight at the transcriptional level. GSDME can be detected in the heart, brain, and tumor cells and has promising research prospects in tumor cells (62, 63). Shao’s research team has found that chemotherapy drugs used in clinical practice can activate GSDME-mediated pyroptosis. Caspase-3 is activated to cut Asp270 of GSDME and induces the occurrence of pyroptosis after chemotherapy drugs, which explains the strong side effects brought to patients after the use of chemotherapy drugs (53, 64). However, studies have found that, compared with normal cells, GSDME is not expressed in most tumor cells. The combination of chemotherapy drugs and GSDME to induce intense pyroptosis of tumor cells and protect normal cells is the key to future research (53). It is reported that iron increases intracellular ROS levels and combined therapy with drugs enhances the therapeutic effect of drugs due to the elevation of ROS production. High levels of ROS promote the release of cytochrome C, which activates caspase-3, and caspase-3 cleaves GSDME, leading to the activation of GSDME and inducing pyroptosis. Researchers have also found that the poor effect of clinical chemotherapy drugs may be related to pyroptosis, and found the unique effect of the iron agent. Combined with clinical chemotherapy drugs, the proliferation and metastasis of tumor cells were inhibited (65). GSDME expression in tumor cells can transform apoptosis into pyroptosis, which provides a way out of the poor efficacy of chemotherapy drugs and overcomes the drug resistance of tumor cells (66). With further research, it was also found that the tumor suppressor p53 activated GSDME through transcription, and GSDME induced the occurrence of cell pyroptosis, outflow of inflammatory factors, and recruitment of macrophages to remove more tumor cells (67).

### GSDMF

GSDMF, unlike other members of the GSDM family, has only one C-terminal domain. GSDMF can be detected in a variety of tissues, but there are few specific studies (68). Like GSDME, GSDMF is associated with hearing loss in humans, and patients with GSDMF mutations present dysfunctions of the cochlear hair cells of the cochlea or auditory neuropathy, which impairs the neurotransmission of auditory signals (69). Excessive noise causes excessive oxidative stress on auditory hair cells and damages the function of auditory hair cells in hearing impairment. GSDMF, as a peroxisome-related protein, degrades peroxisome through rapid, we selective autophagy to protect cells from functional damage during excessive oxidative stress of auditory hair cells (70).

## Caspase Family

Caspases are evolutionarily conserved proteins, which consist of amino-terminal domains and large and small catalytic subunits. Activation of large and small catalytic subunits is also key to the executive function of caspase. Caspases specifically cleave certain substrate proteins, mainly acting on the aspartic acid residues of these proteins. The amino-terminal region of initiator caspases contains the caspases recruitment domain or death effect domain, which promotes their recruitment and activation in the multi-protein complex (76). Executioner caspases, in contrast, lack the amino-terminal domain and its activation is needed to be cleaved, as shown in Table 2. Prior studies have shown that caspases are associated with apoptosis, homeostasis, and an insoluble modulated mode of cell death that supports the removal of senescent and damaged cells (77). Compared with apoptosis, necrosis emerged as a new way of death mediated by Receptor interacting protein kinase (RIPK) and mixed-lineage kinase domain-like protein (MLKL) (78). With the deepening of caspase research, a dependent on inflammasome induced pyroptosis, caspase-1/4/5/11 cleavage GSDMD, induce the emergence of pyroptosis, and caspase-1 can also combine pro-IL-1β and pro-IL-18. The formation of active IL-1β and IL-18, released extracellular by GSDMD pore, leads to an inflammatory response. Previous studies have shown that abnormal regulation of caspase is the mechanism of tumorigenesis, autoimmunity and autoinflammation (79).

**TABLE 2:** Key information of caspase family.

Caspase	Organism	Type	Biological function	References
Caspase-1	Human and mouse	Cytokine Activators	Pyroptosis	(80)
Caspase-2	Human and mouse	Initiator Caspases	RCD	(81)
Caspase-3	Human and mouse	Executioner Caspases	Apoptosis/pyroptosis	(64, 82)
Caspase-4	Human	Cytokine Activators	Pyroptosis	(21, 83)
Caspase-5	Human	Cytokine Activators	Pyroptosis	(11, 83)
Caspase-6	Human and mouse	Executioner Caspases	Apoptotic/pyroptosis	(84)
Caspase-7	Human and mouse	Executioner Caspases	Apoptotic	(85)
Caspase-8	Human and mouse	Initiator Caspases	Necroptosis/pyroptosis	(86–89)
Caspase-9	Human and mouse	Initiator Caspases	RCD	(85)
Caspase-10	Human and mouse	Initiator Caspases	RCD	(90)
Caspase-11	Mouse	Cytokine Activators	Pyroptosis	(21, 22, 91)
Caspase-12	Mouse	Not known	RCD	(92, 93)
Caspase-14	Human and mouse	Executioner Caspases	Unknown	(94–96)

## Inflammasome

The innate immune system relies heavily on the assembly and activation of inflammasome (97), the inflammasome is made up of sensors, adapters, and effectors, the sensors determine the type of inflammasome. The most mature inflammasome was NOD-like receptor protein 3 (NLRP3); NOD-like receptor protein 1 (NLRP1); NLR family inhibitor of apoptosis protein (NAIP); NOD-like receptor protein and CARD domain 4 (NLRC4). What’s more, many other NLR family proteins, including, NLRP6, NLRP7, NLRP12, AIM2, and Pyrin inflammasome, are also thought to be sensors of inflammasome complexes. The nod like receptor (NLR) family of pattern recognition receptors respond to pathogen-related molecules or host-derived injury-related molecules. After activation of the inflammasome, NLR forms oligomerize through its a central nucleotide-binding and oligomerization domain with ATPase activity (NACHT) domain, which promotes caspase-1 recruitment through direct interaction between NLR and caspase-1. However, most NLRs lack the CARD domain and cannot recruit inflammation-related caspases alone. To solve this problem, NLRs recruit (apoptosis-associated speck-like protein containing CARD) ASC, and the Pyrin domain (PYD) domain of ASC interacts with the PYD domain of NLR to recruit caspase-1 through the CARD domain of ASC. Therefore, activating caspase-1 leads to the occurrence and development of pyroptosis (98). NLR can recognize ligands from a variety of microbial pathogens, host cells, and environmental sources. Based on its domain structure, the NLR is subdivided into NLRP and NLRC. NLRP1, NLRP3, and NLRC4 are NLRs that assemble inflammasomes (99).

### NOD-Like Receptor Protein 1

Nucleotide-binding domain (NBD) and leucine-rich repeat sequence (LRR) containing Pyrin domain protein 1 (NLRP1) are inflammatory body sensors that mediate activation of caspase-1 to induce cytokine maturation and pyroptosis (100, 101). NLRP1 contains a functional lookup domain (FIIND) that automatically hydrolyzes proteins into non-covalently related subdomains (102). NLRP1-FL and DPP9 have been shown to act as checkpoints for NLRP1 inflammasome activation by directly binding to isolate inflammatory NLRP1-CT (103).

### NOD-Like Receptor Protein 3

NLRP3 acts as an activator of inflammatory response and contains three domains, leucine-rich repeat (LRR), oligomerization domain with ATPase activity (NACHT), PYD domain (104). Therefore, NLRP3 inflammasome requires initiation and activation processes, during which multiple signaling receptors induce NLRP3 expression. Signals provided by the NF-κB activator are necessary for NLRP3 activation. Moreover, PAMPs or DAMPs are required for the activation of inflammasome (105). PAMPs and DAMPs accelerate the assembly of the NLRP3 inflammasome, which leads to the maturation and release of caspase-1-mediated inflammatory cytokines and pyroptosis (106).

### NOD-Like Receptor Protein and CARD Domain 4

NLRC4 is a nod-like receptor family member expressed in innate immune cells. It indirectly senses bacterial flagellin and III type secretion system and reacts by assembling inflammasome complex to promote caspase-1 activation and pyroptosis (107–109). NAIP-NLRC4 inflammasome is strongly activated in intestinal epithelial cells (IEC) following pathogen infection, resulting in pyroptosis and subsequent expulsion of infected cells into the intestinal.

## Role of Pyroptosis in Cardiovascular Diseases

Cardiovascular disease is a significant cause of death and reduced quality of life worldwide. Studies have shown that pyroptosis is associated with the pathogenesis of many cardiovascular diseases. Endothelial cells pyroptosis (110), macrophages pyroptosis (111) and smooth muscle cells pyroptosis (112) are intimately connected to the emergence and progress of atherosclerotic lesions and the stability of plaques (113). The expression of NLRP3/IL-1β/caspase-1 has been observed in patients with diabetes, myocardial infarction, arrhythmia, and cardiac hypertrophy, and some agents can improve the symptoms of cardiovascular disease in patients by inhibiting the occurrence of pyroptosis. We will describe separately from atherosclerosis, myocardial infarction, diabetes, arrhythmia, and cardiac hypertrophy. The relationship between pyroptosis and cardiovascular disease is shown in Table 3.

**TABLE 3:** Pyroptosis and cardiovascular disease.

Cardiovascular disease	Therapeutic targets	Biological functions	References
Atherosclerosis	NLRP3/ASC/Caspase-1/IL-1β	Reduced expression of VCAM-1 and monocyte chemoattractant protein-1, reducing atherosclerotic plaques	(116–118)
Diabetic cardiomyopathy	IL-1β/NLRP3/Caspase-1	LPS, miR-214-3p and IAPP trigger pyroptosis and induce diabetes	(121, 122, 126)
Acute myocardial infarction	NLRP3/Caspase-1/IL-18	Increased NLRP3/IL-1β/caspase-1 expression and increased myocardial infarction size	(128, 132, 133)
Arrhythmia	Caspase-1/IL-1β/IL-18	Activation of the NLRP3 inflammasome triggers arrhythmias	(139, 140)
Cardiac hypertrophy	NLRP3/IL-18/IL-1β/Caspase-1	Deletion of IL-1β, Tripterygium wilfordii Hook F, SiNPs, and irisin reduces cardiac hypertrophy	(141, 142, 144, 145)

### Pyroptosis in Atherosclerosis

Atherosclerosis is a chronic progressive disease characterized by lipid deposition in the arteries, the formation of plaques leads to vascular stenosis, vascular stenosis caused by atherosclerosis and function of organs in the blood supply can produce a significant effect, plaque rupture, then induce important organs such as heart, brain, kidney embolism phenomenon. In recent years, more and more pieces of evidence suggest that atherosclerosis is an inflammatory disease associated with endothelial dysfunction, and pyroptosis is the key cause of endothelial dysfunction (111, 114, 115). Among them, NLRP3 inflammasome components NLRP3, ASC, caspase-1 are highly expressed in carotid atherosclerotic plaques, suggesting that they are related to the pathogenesis of atherosclerosis (116, 117). The negative effect of NLRP3 on atherosclerosis is mainly dependent on its effector cytokine IL-1β. In IL-1β deficient mice, the area of atherosclerotic plaque is reduced by about 30%, which may indicate that IL-1β deficiency inhibits the migration of monocytes to lipid deposition sites (118, 119). These findings confirm that pyroptosis is involved in the formation of atherosclerotic plaques, and inhibition of NLRP3 and IL-1β in the pyroptosis pathway may be a strategy for the treatment of atherosclerosis.

### Pyroptosis in Diabetic Cardiomyopathy

Cardiomyopathy is a common clinical syndrome in the terminal stage of diabetes mellitus. Cardiac inflammation and oxidative stress are the main causes of diabetes. High glucose induces increased ROS levels in cardiomyocytes, and mitochondrial dysfunction leads to cardiomyocyte death (120). High levels of ROS and inflammasome can also trigger pyroptosis, prompting that pyroptosis plays an irreplaceable part in the occurrence and progress of diabetic cardiomyopathy. Previous studies have shown that islet amyloid polypeptide (IAPP) is a protein formed in patients with diabetes that causes activation of the NLRP3 inflammasome, triggering a series of inflammatory responses that produce mature IL-1β, a process also known as glucose metabolism (121). LPS and IAPP have the same pathological characteristics. It was found that continuous high glucose feeding increased the proportion of intestinal microbiota containing lipopolysaccharide, and the concentration of plasma LPS was upregulated. The upregulation of LPS led to the expression of NLRP3 and IL-1β, thereby inducing the occurrence of diabetes (122). In a mice model of spontaneous non-obese diabetes, activation of NLRP3 promotes the proliferation, differentiation, and transport of diabetic TH1 cells to islets, thereby delaying the onset of diabetes (123). It was found that NLRP3 silencing improved myocardial inflammation, fibrosis, and cardiac dysfunction in type ii diabetic mice induced by high glucose, suggesting that the reduction of pyroptosis can improve the complications of diabetes (124). In addition, similar results were observed in H9c2 cells treated with high glucose. More importantly, LPS was shown to induce pyroptosis through activation of the ROS-dependent NLRP3 inflammasome of H9C2 cardiomyocytes, followed by the NF-κB signaling pathway (125). In diabetic cardiomyopathy, increased levels of caspase-1 related circRNAs activate Caspase-1 activity and cause pyroptosis, while silencing caspase-1 related circRNAs significantly reduces pyroptosis (126). These findings confirm that high levels of ROS, LPS and inflammasome are involved in the development of diabetes, and inhibition of NLRP3 and caspase-1 in the pyroptosis pathway can alleviate diabetes.

### Pyroptosis in Acute Myocardial Infarction

Coronary thrombosis caused by the rupture of unstable atherosclerotic plaque and continuous interruption of blood flow in diseased coronary arteries is the main cause of acute myocardial infarction (ACM). For patients with ACM, the best treatment plan is percutaneous coronary intervention (PCI) to restore coronary blood supply as soon as possible to reduce myocardial cell death (127), the ischemic necrotic myocardium releases a large amount of ATP and oxidative stress products (reactive oxygen species), which are activators of NLRP3 inflammasome. Some studies have reported that loss of NLRP3 inflammasome alleviates inflammatory response and improves cardiac dysfunction induced by myocardial infarction (MI). Therefore, the regulation of NLRP3 inflammasome is considered as a underlying curative mark for MI (128). Studies have shown that MicroRNA-29a inhibits oxidative stress, while NLRP3 ameliorates MI through SIRT1 (129). Intriguingly, colchicine, as a non-specific inhibitor of the NLRP3, was shown to significantly reduce infarct size in phase II clinical trials (130). In 2001, a study showed that inhibiting caspase-1 with an inhibitor restored heart function (131). IL-18 binding protein and IL-1 receptor blockers also had similar protective effects, suggesting that interleukin is involved in mediating injury. To further understand the role of inflammation-related caspase-1 in myocardial ischemia-reperfusion (I/R) injury, we constructed transgenic mice with caspase-1. After I/R surgery, the size of MI increased, whereas caspase-1 knockout mice showed a better therapeutic effect and reduced the size of myocardial infarction (132, 133). Z-vad, a commonly used inhibitor of apoptosis, had the same inhibitory effect on pyroapoptosis and restored the size of MI (134, 135). Another caspase-1 inhibitor, VX-765, also had the same effect, which may be related to the increase of the reperfusion injury salvage kinase (RISK) expression by inhibiting caspase-1 (136). After tail vein injection of VX-765 in MI model mice, the expression of inflammatory and pyroptosis-related factors was decreased, and the infarct size left ventricular remodeling and left ventricular function were decreased (137). These findings confirm that large amounts of ATP and oxidative stress can activate NLRP3, thereby increasing myocardial infarct size, while some inhibitors of oxidative stress can reduce myocardial infarct size by inhibiting the expression of NLRP3 and caspase-1.

### Pyroptosis in Arrhythmia

Atrial fibrillation (AF) is the most common persistent arrhythmia, has a complex mechanism, and electrical remodeling and structural remodeling of atrial muscle are two major pathological bases for its occurrence and maintenance (138). Yao et al. isolated atrial myocytes from atrial tissue of patients with AF and detected the expression of the p20 subunit of caspase-1, and found that the expression of the p20 subunit in atrial myocytes of patients with paroxysmal AF and persistent AF was significantly increased compared with normal controls (139). Studies have shown that oxidative stress and inflammatory response linked in the occurrence of AF, and serum IL-1β and IL-18 levels are positively correlated with AF (140).

### Pyroptosis in Cardiac Hypertrophy

Cardiac hypertrophy is an adaptive response to cardiac pressure overload. Prolonged cardiac overload can lead to cardiovascular disease. The most common causes of cardiac hypertrophy are hypertrophic cardiomyopathy, hypertension, and valvular stenosis. Pyroptosis-related factors play a critical role in the cardiac hypertrophic reaction. IL-18, a myocardial pro-inflammatory cytokine, is raised the serum of sick persons with cardiac hypertrophy, and hypertrophy-related genes are down-regulated in IL-18 knockout mice, suggesting a key role in cardiac hypertrophy (141). In addition to IL-18, leading to cardiac hypertrophy (142). Taken together, NLRP3 inflammasome is concerned with the pathological changes of cardiac hypertrophy, and pirfenidone ameliorates left ventricular hypertrophy in mice with coarctation aorta by forbiding NLRP3 inflammasome combination and adjustment ROS-dependent NLRP3-IL-1β signaling and myocardial fibrosis (143). Studies have shown that silica nanoparticles (SiNPs) are associated with cardiovascular disease. It was found that SiNPs could induce ROS oxidative damage and promote the expression of inflammatory factors. At the same time, SiNPs can up-regulate the expression of cardiac hypertrophy-related genes, and can also promote cardiomyocyte pyroptosis by up-regulating the expression of pyroptosis-related proteins. The NADPH inhibitor VAS2870 can effectively inhibit the level of ROS. The decrease in the level of ROS causes the NLRP3 inflammasome to fail to assemble and activate the caspase-1 signaling pathway, thereby inhibiting pyroptosis and cardiac hypertrophy (144). There are also studies showing that irisin is a promising therapeutic agent for inhibiting nlrp3-mediated cardiomyocyte pyroptosis (145). These findings confirm that in patients with cardiac hypertrophy, ROS levels induce oxidative stress, leading to increased expression of NLRP3/IL-18, and some therapeutic agents associated with oxidative stress can reduce the expression of genes associated with cardiac hypertrophy.

## Conclusion and Outlook

In summary, we can see that the occurrence of inflammation is highly related to cardiovascular disease. Whether it is ischemic heart disease or non-ischemic heart disease, inflammation-related pyroptosis is involved in the occurrence and development of cardiovascular disease. The expressions of pyroptosis-related NLRP3, GSDMD, caspase-1, IL-1β, and IL-18 in these heart disease patients were elevated. With the efforts of many research teams, NLRP3, GSDMD, caspase-1, IL-1β were also confirmed. The key role of IL-18 in cardiomyocyte pyroptosis, necrotic cardiomyocytes can trigger an inflammatory cascade, dead cells release intracellular components, and the NLRP3 inflammasome recognizes PAMPs and DAMPs, thereby further stimulating the innate immune mechanism and promoting inflammation the activation of the NLRP3 inflammasome can activate caspase-1, and the activated caspase-1 induces the occurrence of pyroptosis, and further exerts inflammation by inducing the secretion of pro-inflammatory cytokines (i.e., IL-1β, IL-18). Effect, aggravating cardiovascular disease due to the combined effect of changes in glycolipid energy metabolism, oxidative stress, and systemic inflammatory responses. The current research mainly inhibits the occurrence of pyroptosis by inhibiting the expression of pyroptosis-related proteins or inflammatory factors, and inhibiting the occurrence of pyroptosis does relieve the symptoms of cardiovascular diseases, and also opens up opportunities for the development of drugs for the treatment of cardiovascular diseases in the future. A new way studies have also found that some specific drugs perform well in pyroptosis, including SiNPs, Ac-YVAD-CMK, BAY11-7082, NAC, etc. These drugs also inhibit the occurrence of interactions through the ROS/NLRP3/caspase-1 signaling pathway. Up to now, research has found that everyone has focused on how to inhibit the occurrence of inflammation, including inhibiting the activation of the NLRP3 inflammasome and the activation of caspase-1. Release of IL-1β and IL-18. Of course, IL-1β and IL-18 have great prospects as biomarkers for predicting the occurrence of cardiovascular diseases. IL-1β and IL-18 are relatively stable in serum, and the determination is simple and sensitive. The development of clinical standards and the development of relevant test kits can complement current clinical diagnostic methods.

So far, the application of pyroptosis is still in the basic research stage. The identification methods of pyroptosis mainly focus on the identification of morphology and the detection of pyroptosis-related proteins. In the morphological identification, electron microscope was mainly used to observe the cell morphology. When the cells were pyroptosis, the chromatin was intact, the cells were swollen and swollen, bubbles on the cell membrane, and pyroptosis bodies were produced. Chromatin integrity was detected by TUNEL, pyroptosis-related proteins were detected by Western blot, including caspase-1/4/5/11, GSDMD, IL-1β, IL-18, and the levels of inflammatory factors could also be detected by ELISA kits, including IL-1β, IL-18. In addition, the expression of GSDMD can also be observed by immunofluorescence. With the improvement of the mechanism of pyroptosis, more and more researchers realize that pyroptosis plays an important role in the occurrence and development of cardiovascular diseases. Although pyroptosis has shown great promise as a therapeutic target for cardiovascular disease, its clinical application still has a long way to go due to diagnostic limitations. Finding biomarkers of cardiovascular disease is an important means to prevent disease, and the three most important features of biomarkers are specificity, sensitivity, and stability. GSDM protein and inflammatory factors in pyroptosis can be used as potential biomarkers, because they are stable (i.e., unaffected by ribonucleases) and can be used simply and sensitively in blood, or other body fluids, must be standardized clinically. Therefore, it is necessary to further validate the function of pyroptosis and integrate it into clinical practice. For example, (1) standardized methods need to be developed to detect pyroptosis levels in body fluids and tissues; (2) further analysis of optimal blood components for pyroptosis detection is beneficial for the development of clinical pyroptosis diagnosis; (3) The development of pyroptosis inhibitors is beneficial to the development of cardiovascular clinical drugs. In summary, the mechanism of pyroptosis and its molecular mechanism of action in cardiovascular disease remain to be elucidated, which will provide new theoretical basis and analytical ideas for the diagnosis and treatment of cardiovascular disease.

## Author Contributions

XC and P-CT reviewed the literature and drafted the manuscript. KaW designed the figures. MW and KuW provided supervision and revised the manuscript. All authors jointly conceptualized the article and approved the final manuscript.

## Conflict of Interest

The authors declare that the research was conducted in the absence of any commercial or financial relationships that could be construed as a potential conflict of interest.

## Publisher’s Note

All claims expressed in this article are solely those of the authors and do not necessarily represent those of their affiliated organizations, or those of the publisher, the editors and the reviewers. Any product that may be evaluated in this article, or claim that may be made by its manufacturer, is not guaranteed or endorsed by the publisher.

## References

[1] Segawa K Nagata S. An apoptotic ‘Eat Me’ signal: phosphatidylserine exposure. *Trends Cell Biol.* (2015) 25:639–50. 10.1016/j.tcb.2015.08.003 26437594

[2] Galluzzi L Vitale I Aaronson SA Abrams JM Adam D Agostinis P Molecular mechanisms of cell death: recommendations of the nomenclature committee on cell death 2018. *Cell Death Differ.* (2018) 25:486–541. 10.1038/s41418-017-0012-4 29362479 PMC5864239

[3] Dixon SJ Lemberg KM Lamprecht MR Skouta R Zaitsev EM Gleason CE Ferroptosis: an iron-dependent form of nonapoptotic cell death. *Cell.* (2012) 149:1060–72. 10.1016/j.cell.2012.03.042 22632970 PMC3367386

[4] Song X Zhu S Xie Y Liu J Sun L Zeng D JTC801 Induces pH-dependent death specifically in cancer cells and slows growth of tumors in mice. *Gastroenterology.* (2018) 154:1480–93. 10.1053/j.gastro.2017.12.004 29248440 PMC5880694

[5] Fink SL Cookson BT. Apoptosis, pyroptosis, and necrosis: mechanistic description of dead and dying eukaryotic cells. *Infect Immun.* (2005) 73:1907–16. 10.1128/iai.73.4.1907-1916.2005 15784530 PMC1087413

[6] Cookson BT Brennan MA. Pro-inflammatory programmed cell death. *Trends Microbiol.* (2001) 9:113–4. 10.1016/s0966-842x(00)01936-3 11303500

[7] Zychlinsky A Prevost MC Sansonetti PJ. *Shigella flexneri* induces apoptosis in infected macrophages. *Nature.* (1992) 358:167–9. 10.1038/358167a0 1614548

[8] Zychlinsky A Fitting C Cavaillon JM Sansonetti PJ. Interleukin 1 is released by murine macrophages during apoptosis induced by *Shigella flexneri*. *J Clin Invest.* (1994) 94:1328–32. 10.1172/jci117452 8083373 PMC295219

[9] Chen Y Smith MR Thirumalai K Zychlinsky AA. Bacterial invasin induces macrophage apoptosis by binding directly to ice. *EMBO J.* (1996) 15:3853–60. 10.1002/j.1460-2075.1996.tb00759.x 8670890 PMC452076

[10] Kayagaki N Stowe IB Lee BL O’Rourke K Anderson K Warming S Caspase-11 cleaves gasdermin D for non-canonical inflammasome signalling. *Nature.* (2015) 526:666–71. 10.1038/nature15541 26375259

[11] Shi J Zhao Y Wang K Shi X Wang Y Huang H Cleavage of GSDMD by inflammatory caspases determines pyroptosis cell death. *Nature.* (2015) 526:660–5. 10.1038/nature15514 26375003

[12] Liu X Zhang Z Ruan J Pan Y Magupalli VG Wu H Inflammasome-activated gasdermin D causes pyroptosis by forming membrane pores. *Nature.* (2016) 535:153–8. 10.1038/nature18629 27383986 PMC5539988

[13] Lamkanfi M Sarkar A Vande Walle L Vitari AC Amer AO Wewers MD inflammasome-dependent release of the alarmin HMGB1 in endotoxemia. *J Immunol.* (2010) 185:4385–92. 10.4049/jimmunol.1000803 20802146 PMC3428148

[14] von Moltke J Trinidad NJ Moayeri M Kintzer AF Wang SB van Rooijen N Rapid induction of inflammatory lipid mediators by the inflammasome in vivo. *Nature.* (2012) 490:107–11. 10.1038/nature11351 22902502 PMC3465483

[15] Gaidt MM Ebert TS Chauhan D Schmidt T Schmid-Burgk JL Rapino F Human monocytes engage an alternative inflammasome pathway. *Immunity.* (2016) 44:833–46. 10.1016/j.immuni.2016.01.012 27037191

[16] Evavold CL Ruan J Tan Y Xia S Wu H Kagan JC. The pore-forming protein gasdermin D regulates interleukin-1 secretion from living macrophages. *Immunity.* (2018) 48:35–44.e6. 10.1016/j.immuni.2017.11.013 29195811 PMC5773350

[17] Chen KW Groß CJ Sotomayor FV Stacey KJ Tschopp J Sweet MJ The neutrophil NLRC4 inflammasome selectively promotes Il-1β maturation without pyroptosis during acute *Salmonella* Challenge. *Cell Rep.* (2014) 8:570–82. 10.1016/j.celrep.2014.06.028 25043180

[18] Jorgensen I Zhang Y Krantz BA Miao EA. Pyroptosis triggers pore-induced intracellular traps (Pits) that capture bacteria and lead to their clearance by efferocytosis. *J Exp Med.* (2016) 213:2113–28. 10.1084/jem.20151613 27573815 PMC5030797

[19] Jorgensen I Lopez JP Laufer SA Miao EA. Il-1β, Il-18, and eicosanoids promote neutrophil recruitment to pore-induced intracellular traps following pyroptosis. *Eur J Immunol.* (2016) 46:2761–6. 10.1002/eji.201646647 27682622 PMC5138142

[20] Broz P Dixit VM. Inflammasomes: mechanism of assembly, regulation and signalling. *Nature Rev Immunol.* (2016) 16:407–20. 10.1038/nri.2016.58 27291964

[21] Shi J Zhao Y Wang Y Gao W Ding J Li P Inflammatory caspases are innate immune receptors for intracellular LPS. *Nature.* (2014) 514:187–92. 10.1038/nature13683 25119034

[22] Kayagaki N Wong MT Stowe IB Ramani SR Gonzalez LC Akashi-Takamura S Noncanonical inflammasome activation by intracellular LPS independent of TLR4. *Science.* (2013) 341:1246–9. 10.1126/science.1240248 23887873

[23] Li Z Liu W Fu J Cheng S Xu Y Wang Z *Shigella* evades pyroptosis by arginine ADP-riboxanation of caspase-11. *Nature.* (2021) 599:290–5. 10.1038/s41586-021-04020-1 34671164

[24] Tamura M Tanaka S Fujii T Aoki A Komiyama H Ezawa K Members of a novel gene family, Gsdm, are expressed exclusively in the epithelium of the skin and gastrointestinal tract in a highly tissue-specific manner. *Genomics.* (2007) 89:618–29. 10.1016/j.ygeno.2007.01.003 17350798

[25] Li S Wu Y Yang D Wu C Ma C Liu X Gasdermin D in peripheral myeloid cells drives neuroinflammation in experimental autoimmune encephalomyelitis. *J Exp Med.* (2019) 216:2562–81. 10.1084/jem.20190377 31467036 PMC6829591

[26] Xiao J Wang C Yao JC Alippe Y Xu C Kress D Gasdermin D mediates the pathogenesis of neonatal-onset multisystem inflammatory disease in mice. *PLoS Biol.* (2018) 16:e3000047. 10.1371/journal.pbio.3000047 30388107 PMC6235378

[27] Kumar S Rathkolb B Budde BS Nürnberg P de Angelis MH Aigner B GSDMA3(I359N) is a novel ENU-induced mutant mouse line for studying the function of gasdermin A3 in the hair follicle and epidermis. *J Dermatol Sci.* (2012) 67:190–2. 10.1016/j.jdermsci.2012.05.001 22682752

[28] Das S Miller M Beppu AK Mueller J McGeough MD Vuong C GSDMB induces an asthma phenotype characterized by increased airway responsiveness and remodeling without lung inflammation. *Proc Natl Acad Sci USA.* (2016) 113:13132–7. 10.1073/pnas.1610433113 27799535 PMC5135378

[29] Yu C Meng X Zhang S Zhao G Hu L Kong X. A 3-nucleotide deletion in the polypyrimidine tract of intron 7 of the DFNA5 gene causes nonsyndromic hearing impairment in a chinese family. *Genomics.* (2003) 82:575–9. 10.1016/s0888-7543(03)00175-7 14559215

[30] Thurston TL Matthews SA Jennings E Alix E Shao F Shenoy AR Growth inhibition of cytosolic *Salmonella* by caspase-1 and caspase-11 precedes host cell death. *Nat Commun.* (2016) 7:13292. 10.1038/ncomms13292 27808091 PMC5097160

[31] Rathkey JK Zhao J Liu Z Chen Y Yang J Kondolf HC Chemical disruption of the pyroptosis pore-forming protein gasdermin D inhibits inflammatory cell death and sepsis. *Sci Immunol.* (2018) 3:eaat2738. 10.1126/sciimmunol.aat2738 30143556 PMC6462819

[32] Moussette S Al Tuwaijri A Kohan-Ghadr HR Elzein S Farias R Bérubé J Role of DNA methylation in expression control of the IKZF3-GSDMA region in human epithelial cells. *PLoS One.* (2017) 12:e0172707. 10.1371/journal.pone.0172707 28241063 PMC5328393

[33] Croes L Beyens M Fransen E Ibrahim J Vanden Berghe W Suls A Large-scale analysis of DFNA5 methylation reveals its potential as biomarker for breast cancer. *Clin Epigenet.* (2018) 10:51. 10.1186/s13148-018-0479-y 29682089 PMC5896072

[34] Sato H Koide T Masuya H Wakana S Sagai T Umezawa A A new mutation RIM3 resembling Re(Den) is mapped close to retinoic acid receptor alpha (Rara) gene on mouse chromosome 11. *Mamm Genome.* (1998) 9:20–5. 10.1007/s003359900673 9434940

[35] Lei M Bai X Yang T Lai X Qiu W Yang L GSDMA3 is a new factor needed for TNF-α-mediated apoptosis signal pathway in mouse skin keratinocytes. *Histochem Cell Biol.* (2012) 138:385–96. 10.1007/s00418-012-0960-1 22585037

[36] Söderman J Berglind L Almer S. Gene expression-genotype analysis implicates GSDMA, GSDMB, and LRRC3C as contributors to inflammatory bowel disease susceptibility. *Biomed Res Int.* (2015) 2015:834805. 10.1155/2015/834805 26484354 PMC4592899

[37] Yu J Kang MJ Kim BJ Kwon JW Song YH Choi WA Polymorphisms in GSDMA and GSDMB are associated with asthma susceptibility, atopy and BHR. *Pediatr Pulmonol.* (2011) 46:701–8. 10.1002/ppul.21424 21337730

[38] Moreno-Moral A Bagnati M Koturan S Ko JH Fonseca C Harmston N Changes in macrophage transcriptome associate with systemic sclerosis and mediate GSDMA contribution to disease risk. *Ann Rheumat Dis.* (2018) 77:596–601. 10.1136/annrheumdis-2017-212454 29348297 PMC5890626

[39] Saeki N Usui T Aoyagi K Kim DH Sato M Mabuchi T Distinctive expression and function of four GSDM family genes (GSDMA-D) in normal and malignant upper gastrointestinal epithelium. *Genes Chromosomes Cancer.* (2009) 48:261–71. 10.1002/gcc.20636 19051310

[40] Carl-McGrath S Schneider-Stock R Ebert M Röcken C. Differential expression and localisation of gasdermin-like (GSDML), a Novel Member of the Cancer-Associated GSDMDC protein family, in neoplastic and non-neoplastic gastric, hepatic, and colon tissues. *Pathology.* (2008) 40:13–24. 10.1080/00313020701716250 18038310

[41] Hu Y Jin S Cheng L Liu G Jiang Q. Autoimmune disease variants regulate GSDMB gene expression in human immune cells and whole blood. *Proc Natl Acad Sci USA.* (2017) 114:E7860–2. 10.1073/pnas.1712127114 28882878 PMC5617311

[42] Chen Q Shi P Wang Y Zou D Wu X Wang D Gsdmb Promotes Non-Canonical Pyroptosis by Enhancing Caspase-4 Activity. *J Mol Cell Biol.* (2019) 11:496–508. 10.1093/jmcb/mjy056 30321352 PMC6734491

[43] Verlaan DJ Berlivet S Hunninghake GM Madore AM Larivière M Moussette S Allele-specific chromatin remodeling in the ZPBP2/GSDMB/ORMDL3 locus associated with the risk of asthma and autoimmune disease. *Am J Hum Genet.* (2009) 85:377–93. 10.1016/j.ajhg.2009.08.007 19732864 PMC2771592

[44] Moffatt MF Gut IG Demenais F Strachan DP Bouzigon E Heath S A large-scale, consortium-based genomewide association study of asthma. *N Engl J Med.* (2010) 363:1211–21. 10.1056/NEJMoa0906312 20860503 PMC4260321

[45] Hergueta-Redondo M Sarrió D Molina-Crespo Á Megias D Mota A Rojo-Sebastian A Gasdermin-B promotes invasion and metastasis in breast cancer cells. *PLoS One.* (2014) 9:e90099. 10.1371/journal.pone.0090099 24675552 PMC3967990

[46] Sun Q Yang J Xing G Sun Q Zhang L He F. Expression of GSDML associates with tumor progression in uterine cervix cancer. *Transl Oncol.* (2008) 1:73–83. 10.1593/tlo.08112 18633457 PMC2510814

[47] Hergueta-Redondo M Sarrio D Molina-Crespo Á Vicario R Bernadó-Morales C Martínez L Gasdermin B expression predicts poor clinical outcome in HER2-positive breast cancer. *Oncotarget.* (2016) 7:56295–308. 10.18632/oncotarget.10787 27462779 PMC5302915

[48] Katoh M Katoh M. Identification and characterization of human DFNA5L, mouse DFNA5L, and rat DFNA5L genes in silico. *Int J Oncol.* (2004) 25:765–70. 15289881

[49] Miguchi M Hinoi T Shimomura M Adachi T Saito Y Niitsu H Gasdermin C is upregulated by inactivation of transforming growth factor B receptor type II in the presence of mutated APC, promoting colorectal cancer proliferation. *PLoS One.* (2016) 11:e0166422. 10.1371/journal.pone.0166422 27835699 PMC5105946

[50] Zhang JY Zhou B Sun RY Ai YL Cheng K Li FN The metabolite A -KG induces GSDMC-dependent pyroptosis through death receptor 6-activated caspase-8. *Cell Res.* (2021) 31:980–97. 10.1038/s41422-021-00506-9 34012073 PMC8410789

[51] Hou J Zhao R Xia W Chang CW You Y Hsu JM PD-L1-mediated gasdermin C expression switches apoptosis to pyroptosis in cancer cells and facilitates tumour necrosis. *Nat Cell Biol.* (2020) 22:1264–75. 10.1038/s41556-020-0575-z 32929201 PMC7653546

[52] Fujii T Tamura M Tanaka S Kato Y Yamamoto H Mizushina Y Gasdermin D (GSDMD) is dispensable for mouse intestinal epithelium development. *Genesis.* (2008) 46:418–23. 10.1002/dvg.20412 18693275

[53] Rogers C Fernandes-Alnemri T Mayes L Alnemri D Cingolani G Alnemri ES. Cleavage of DFNA5 by caspase-3 during apoptosis mediates progression to secondary necrotic/pyroptosis cell death. *Nat Commun.* (2017) 8:14128. 10.1038/ncomms14128 28045099 PMC5216131

[54] Yang X Cheng X Tang Y Qiu X Wang Y Kang H Bacterial endotoxin activates the coagulation cascade through gasdermin D-dependent phosphatidylserine exposure. *Immunity.* (2019) 51:983–96.e6. 10.1016/j.immuni.2019.11.005 31836429

[55] Wu C Lu W Zhang Y Zhang G Shi X Hisada Y Inflammasome activation triggers blood clotting and host death through pyroptosis. *Immunity.* (2019) 50:1401–11.e4. 10.1016/j.immuni.2019.04.003 31076358 PMC6791531

[56] McDonald B Davis RP Kim SJ Tse M Esmon CT Kolaczkowska E Platelets and neutrophil extracellular traps collaborate to promote intravascular coagulation during sepsis in mice. *Blood.* (2017) 129:1357–67. 10.1182/blood-2016-09-741298 28073784 PMC5345735

[57] Xu B Jiang M Chu Y Wang W Chen D Li X Gasdermin D plays a key role as a pyroptosis executor of non-alcoholic steatohepatitis in humans and mice. *J Hepatol.* (2018) 68:773–82. 10.1016/j.jhep.2017.11.040 29273476

[58] Khanova E Wu R Wang W Yan R Chen Y French SW Pyroptosis by caspase11/4-gasdermin-D pathway in alcoholic hepatitis in mice and patients. *Hepatology.* (2018) 67:1737–53. 10.1002/hep.29645 29108122 PMC5906140

[59] Yang C Sun P Deng M Loughran P Li W Yi Z Gasdermin D protects against noninfectious liver injury by regulating apoptosis and necroptosis. *Cell Death Dis.* (2019) 10:481. 10.1038/s41419-019-1719-6 31209224 PMC6579760

[60] Li J Zhao J Xu M Li M Wang B Qu X Blocking GSDMD processing in innate immune cells but not in hepatocytes protects hepatic ischemia-reperfusion injury. *Cell Death Dis.* (2020) 11:244. 10.1038/s41419-020-2437-9 32303674 PMC7165177

[61] Van Laer L Huizing EH Verstreken M van Zuijlen D Wauters JG Bossuyt PJ Nonsyndromic hearing impairment is associated with a mutation in DFNA5. *Nat Genet.* (1998) 20:194–7. 10.1038/2503 9771715

[62] de Beeck KO Van Laer L Van Camp G. DFNA5, a gene involved in hearing loss and cancer: a review. *Ann Otol Rhinol Laryngol.* (2012) 121:197–207. 10.1177/000348941212100310 22530481

[63] Thompson DA Weigel RJ. Characterization of a gene that is inversely correlated with estrogen receptor expression (ICERE-1) in breast carcinomas. *Eur J Biochem.* (1998) 252:169–77. 10.1046/j.1432-1327.1998.2520169.x 9523727

[64] Wang Y Gao W Shi X Ding J Liu W He H Chemotherapy drugs induce pyroptosis through caspase-3 cleavage of a gasdermin. *Nature.* (2017) 547:99–103. 10.1038/nature22393 28459430

[65] Zhou B Zhang JY Liu XS Chen HZ Ai YL Cheng K Tom20 senses iron-activated ROS signaling to promote melanoma cell pyroptosis. *Cell Res.* (2018) 28:1171–85. 10.1038/s41422-018-0090-y 30287942 PMC6274649

[66] Zhang Z Zhang Y Xia S Kong Q Li S Liu X Gasdermin E suppresses tumour growth by activating anti-tumour immunity. *Nature.* (2020) 579:415–20. 10.1038/s41586-020-2071-9 32188940 PMC7123794

[67] Masuda Y Futamura M Kamino H Nakamura Y Kitamura N Ohnishi S The potential role of DFNA5, a hearing impairment gene, in P53-mediated cellular response to DNA damage. *J Hum Genet.* (2006) 51:652–64. 10.1007/s10038-006-0004-6 16897187

[68] Delmaghani S Defourny J Aghaie A Beurg M Dulon D Thelen N Hypervulnerability to sound exposure through impaired adaptive proliferation of peroxisomes. *Cell.* (2015) 163:894–906. 10.1016/j.cell.2015.10.023 26544938

[69] Schwander M Sczaniecka A Grillet N Bailey JS Avenarius M Najmabadi H a forward genetics screen in mice identifies recessive deafness traits and reveals that pejvakin is essential for outer hair cell function. *J Neurosci.* (2007) 27:2163–75. 10.1523/jneurosci.4975-06.2007 17329413 PMC6673480

[70] Defourny J Aghaie A Perfettini I Avan P Delmaghani S Petit C. Pejvakin-mediated pexophagy protects auditory hair cells against noise-induced damage. *Proc Natl Acad Sci USA.* (2019) 116:8010–7. 10.1073/pnas.1821844116 30936319 PMC6475433

[71] Runkel F Marquardt A Stoeger C Kochmann E Simon D Kohnke B The dominant alopecia phenotypes bareskin, rex-denuded, and reduced coat 2 are caused by mutations in gasdermin 3. *Genomics.* (2004) 84:824–35. 10.1016/j.ygeno.2004.07.003 15475261

[72] Zhou Z He H Wang K Shi X Wang Y Su Y Granzyme a from cytotoxic lymphocytes cleaves GSDMB to trigger pyroptosis in target cells. *Science.* (2020) 368:eaaz7548. 10.1126/science.aaz7548 32299851

[73] Sarhan J Liu BC Muendlein HI Li P Nilson R Tang AY Caspase-8 induces cleavage of gasdermin D to elicit pyroptosis during yersinia infection. *Proc Natl Acad Sci USA.* (2018) 115:E10888–97. 10.1073/pnas.1809548115 30381458 PMC6243247

[74] Kanneganti A Malireddi RKS Saavedra PHV Vande Walle L Van Gorp H Kambara H GSDMD is critical for autoinflammatory pathology in a mouse model of familial mediterranean fever. *J Exp Med.* (2018) 215:1519–29. 10.1084/jem.20172060 29793924 PMC5987922

[75] Delmaghani S del Castillo FJ Michel V Leibovici M Aghaie A Ron U Mutations in the gene encoding pejvakin, a newly identified protein of the afferent auditory pathway, cause DFNB59 auditory neuropathy. *Nat Genet.* (2006) 38:770–8. 10.1038/ng1829 16804542

[76] Lamkanfi M Declercq W Kalai M Saelens X Vandenabeele P. Alice in caspase land. a phylogenetic analysis of caspases from worm to man. *Cell Death Differ.* (2002) 9:358–61. 10.1038/sj.cdd.4400989 11965488

[77] Ramirez MLG Salvesen GSA. Primer on caspase mechanisms. *Semin Cell Dev Biol.* (2018) 82:79–85. 10.1016/j.semcdb.2018.01.002 29329946 PMC6043420

[78] Pasparakis M Vandenabeele P. Necroptosis and its role in inflammation. *Nature.* (2015) 517:311–20. 10.1038/nature14191 25592536

[79] Van Gorp H Van Opdenbosch N Lamkanfi M. Inflammasome-dependent cytokines at the crossroads of health and autoinflammatory disease. *Cold Spring Harb Perspect Biol.* (2019) 11:a028563. 10.1101/cshperspect.a028563 29038114 PMC6314066

[80] Wang K Sun Q Zhong X Zeng M Zeng H Shi X Structural mechanism for GSDMD targeting by autoprocessed caspases in pyroptosis. *Cell.* (2020) 180:941–55.e20. 10.1016/j.cell.2020.02.002 32109412

[81] Bronner DN Abuaita BH Chen X Fitzgerald KA Nuñez G He Y Endoplasmic reticulum stress activates the inflammasome via NLRP3- and caspase-2-driven mitochondrial damage. *Immunity.* (2015) 43:451–62. 10.1016/j.immuni.2015.08.008 26341399 PMC4582788

[82] Huang Q Li F Liu X Li W Shi W Liu FF Caspase 3-mediated stimulation of tumor cell repopulation during cancer radiotherapy. *Nat Med.* (2011) 17:860–6. 10.1038/nm.2385 21725296 PMC3132290

[83] Baker PJ Boucher D Bierschenk D Tebartz C Whitney PG D’Silva DB NLRP3 inflammasome activation downstream of cytoplasmic LPS recognition by both caspase-4 and caspase-5. *Eur J Immunol.* (2015) 45:2918–26. 10.1002/eji.201545655 26173988

[84] Zheng M Karki R Vogel P Kanneganti TD. Caspase-6 is a key regulator of innate immunity, inflammasome activation, and host defense. *Cell.* (2020) 181:674–87.e13. 10.1016/j.cell.2020.03.040 32298652 PMC7425208

[85] Brentnall M Rodriguez-Menocal L De Guevara RL Cepero E Boise LH. Caspase-9, caspase-3 and caspase-7 have distinct roles during intrinsic apoptosis. *BMC Cell Biol.* (2013) 14:32. 10.1186/1471-2121-14-32 23834359 PMC3710246

[86] Kang TB Yang SH Toth B Kovalenko A Wallach D. Caspase-8 blocks kinase RIPK3-mediated activation of the NLRP3 inflammasome. *Immunity.* (2013) 38:27–40. 10.1016/j.immuni.2012.09.015 23260196

[87] Kang S Fernandes-Alnemri T Rogers C Mayes L Wang Y Dillon C Caspase-8 scaffolding function and MLKL regulate NLRP3 inflammasome activation downstream of TLR3. *Nat Commun.* (2015) 6:7515. 10.1038/ncomms8515 26104484 PMC4480782

[88] Panaretakis T Kepp O Brockmeier U Tesniere A Bjorklund AC Chapman DC Mechanisms of pre-apoptotic calreticulin exposure in immunogenic cell death. *EMBO J.* (2009) 28:578–90. 10.1038/emboj.2009.1 19165151 PMC2657583

[89] Tummers B Green DR. Caspase-8: regulating life and death. *Immunol Rev.* (2017) 277:76–89. 10.1111/imr.12541 28462525 PMC5417704

[90] Milhas D Cuvillier O Therville N Clavé P Thomsen M Levade T Caspase-10 Triggers Bid cleavage and caspase cascade activation in FasL-induced apoptosis. *J Biol Chem.* (2005) 280:19836–42. 10.1074/jbc.M414358200 15772077

[91] Kayagaki N Warming S Lamkanfi M Vande Walle L Louie S Dong J Non-canonical inflammasome activation targets caspase-11. *Nature.* (2011) 479:117–21. 10.1038/nature10558 22002608

[92] Saleh M Mathison JC Wolinski MK Bensinger SJ Fitzgerald P Droin N Enhanced bacterial clearance and sepsis resistance in caspase-12-deficient mice. *Nature.* (2006) 440:1064–8. 10.1038/nature04656 16625199

[93] Wang P Arjona A Zhang Y Sultana H Dai J Yang L Caspase-12 controls west nile virus infection via the viral RNA receptor RIG-I. *Nat Immunol.* (2010) 11:912–9. 10.1038/ni.1933 20818395 PMC3712356

[94] Ballaun C Karner S Mrass P Mildner M Buchberger M Bach J Transcription of the caspase-14 gene in human epidermal keratinocytes requires AP-1 and NFKappaB. *Biochem Biophys Res Commun.* (2008) 371:261–6. 10.1016/j.bbrc.2008.04.050 18424262

[95] Bayo P Sanchis A Bravo A Cascallana JL Buder K Tuckermann J Glucocorticoid receptor is required for skin barrier competence. *Endocrinology.* (2008) 149:1377–88. 10.1210/en.2007-0814 18039792

[96] Kubica M Hildebrand F Brinkman BM Goossens D Del Favero J Vercammen K The skin microbiome of caspase-14-deficient mice shows mild dysbiosis. *Exp Dermatol.* (2014) 23:561–7. 10.1111/exd.12458 24863253

[97] Evavold CL Kagan JC. How inflammasomes inform adaptive immunity. *J Mol Biol.* (2018) 430:217–37. 10.1016/j.jmb.2017.09.019 28987733 PMC5766381

[98] de Alba E. Structure and interdomain dynamics of apoptosis-associated speck-like protein containing a CARD (ASC). *J Biol Chem.* (2009) 284:32932–41. 10.1074/jbc.M109.024273 19759015 PMC2781708

[99] Sharma D Kanneganti TD. The cell biology of inflammasomes: mechanisms of inflammasome activation and regulation. *J Cell Biol.* (2016) 213:617–29. 10.1083/jcb.201602089 27325789 PMC4915194

[100] Shen C Sharif H Xia S Wu H. Structural and mechanistic elucidation of inflammasome signaling by cryo-EM. *Curr Opin Struct Biol.* (2019) 58:18–25. 10.1016/j.sbi.2019.03.033 31128494 PMC6778493

[101] Mitchell PS Sandstrom A Vance RE. The NLRP1 inflammasome: new mechanistic insights and unresolved mysteries. *Curr Opin Immunol.* (2019) 60:37–45. 10.1016/j.coi.2019.04.015 31121538 PMC6800612

[102] Finger JN Lich JD Dare LC Cook MN Brown KK Duraiswami C Autolytic proteolysis within the function to find domain (FIIND) is required for nlrp1 inflammasome activity. *J Biol Chem.* (2012) 287:25030–7. 10.1074/jbc.M112.378323 22665479 PMC3408201

[103] Hollingsworth LR Sharif H Griswold AR Fontana P Mintseris J Dagbay KB DPP9 sequesters the C terminus of NLRP1 to repress inflammasome activation. *Nature.* (2021) 592:778–83. 10.1038/s41586-021-03350-4 33731932 PMC8299537

[104] Malik A Kanneganti TD. Inflammasome activation and assembly at a glance. *J Cell Sci.* (2017) 130:3955–63. 10.1242/jcs.207365 29196474 PMC5769591

[105] Bauernfeind FG Horvath G Stutz A Alnemri ES MacDonald K Speert D Cutting edge: nf-kappab activating pattern recognition and cytokine receptors license NLRP3 inflammasome activation by regulating NLRP3 expression. *J Immunol.* (2009) 183:787–91. 10.4049/jimmunol.0901363 19570822 PMC2824855

[106] Christgen S Place DE Kanneganti TD. Toward targeting inflammasomes: insights into their regulation and activation. *Cell Res.* (2020) 30:315–27. 10.1038/s41422-020-0295-8 32152420 PMC7118104

[107] Mariathasan S Newton K Monack DM Vucic D French DM Lee WP Differential activation of the inflammasome by caspase-1 Adaptors ASC and Ipaf. *Nature.* (2004) 430:213–8. 10.1038/nature02664 15190255

[108] Miao EA Mao DP Yudkovsky N Bonneau R Lorang CG Warren SE Innate immune detection of the type III secretion apparatus through the NLRC4 inflammasome. *Proc Natl Acad Sci USA.* (2010) 107:3076–80. 10.1073/pnas.0913087107 20133635 PMC2840275

[109] Zhao Y Yang J Shi J Gong YN Lu Q Xu H The NLRC4 inflammasome receptors for bacterial flagellin and type iii secretion apparatus. *Nature.* (2011) 477:596–600. 10.1038/nature10510 21918512

[110] Long Y Liu X Tan XZ Jiang CX Chen SW Liang GN Ros-induced NLRP3 inflammasome priming and activation mediate PCB 118- induced pyroptosis in endothelial cells. *Ecotoxicol Environ Saf.* (2020) 189:109937. 10.1016/j.ecoenv.2019.109937 31785945

[111] Han Y Qiu H Pei X Fan Y Tian H Geng J. Low-dose sinapic acid abates the pyroptosis of macrophages by downregulation of lncrna-malat1 in rats with diabetic atherosclerosis. *J Cardiovasc Pharmacol.* (2018) 71:104–12. 10.1097/fjc.0000000000000550 29095793

[112] Li Y Niu X Xu H Li Q Meng L He M VX-765 attenuates atherosclerosis in ApoE deficient mice by modulating vsmcs pyroptosis. *Exp Cell Res.* (2020) 389:111847. 10.1016/j.yexcr.2020.111847 31972218

[113] Jia C Chen H Zhang J Zhou K Zhuge Y Niu C Role of pyroptosis in cardiovascular diseases. *Int Immunopharmacol.* (2019) 67:311–8. 10.1016/j.intimp.2018.12.028 30572256

[114] Zhang Y Liu X Bai X Lin Y Li Z Fu J Melatonin prevents endothelial cell pyroptosis via regulation of long noncoding RNA Meg3/mir-223/NLRP3 axis. *J Pineal Res.* (2018) 64:e12448. 10.1111/jpi.12449 29024030

[115] Zhou W Chen C Chen Z Liu L Jiang J Wu Z NLRP3: a novel mediator in cardiovascular disease. *J Immunol Res.* (2018) 2018:5702103. 10.1155/2018/5702103 29850631 PMC5911339

[116] Shi X Xie WL Kong WW Chen D Qu P. Expression of the NLRP3 Inflammasome in Carotid Atherosclerosis. *J Stroke Cerebrovasc Dis.* (2015) 24:2455–66. 10.1016/j.jstrokecerebrovasdis.2015.03.024 26381780

[117] Menu P Pellegrin M Aubert JF Bouzourene K Tardivel A Mazzolai L Atherosclerosis in ApoE-deficient mice progresses independently of the NLRP3 inflammasome. *Cell Death Dis.* (2011) 2:e137. 10.1038/cddis.2011.18 21451572 PMC3101814

[118] Kirii H Niwa T Yamada Y Wada H Saito K Iwakura Y Lack of interleukin-1beta decreases the severity of atherosclerosis in ApoE-deficient mice. *Arterioscler Thromb Vasc Biol.* (2003) 23:656–60. 10.1161/01.Atv.0000064374.15232.C3 12615675

[119] Alexander MR Moehle CW Johnson JL Yang Z Lee JK Jackson CL Genetic inactivation of il-1 signaling enhances atherosclerotic plaque instability and reduces outward vessel remodeling in advanced atherosclerosis in mice. *J Clin Invest.* (2012) 122:70–9. 10.1172/jci43713 22201681 PMC3248279

[120] Guo Y Zhuang X Huang Z Zou J Yang D Hu X Klotho protects the heart from hyperglycemia-induced injury by inactivating ROS and Nf-K b-mediated inflammation both in vitro and in vivo. *Biochim Biophys Acta Mol Basis Dis.* (2018) 1864:238–51. 10.1016/j.bbadis.2017.09.029 28982613

[121] Masters SL Dunne A Subramanian SL Hull RL Tannahill GM Sharp FA Activation of the NLRP3 inflammasome by islet amyloid polypeptide provides a mechanism for enhanced il-1β in type 2 diabetes. *Nat Immunol.* (2010) 11:897–904. 10.1038/ni.1935 20835230 PMC3103663

[122] Saito T Hayashida H Furugen R. Comment On: Cani et al. (2007) Metabolic endotoxemia initiates obesity and insulin resistance: diabetes 56:1761-1772. *Diabetes.* (2007) 56:e20. 10.2337/db07-1181 17456850

[123] Hu C Ding H Li Y Pearson JA Zhang X Flavell RA NLRP3 deficiency protects from type 1 diabetes through the regulation of chemotaxis into the pancreatic islets. *Proc Natl Acad Sci USA.* (2015) 112:11318–23. 10.1073/pnas.1513509112 26305961 PMC4568693

[124] Luo B Li B Wang W Liu X Xia Y Zhang C NLRP3 gene silencing ameliorates diabetic cardiomyopathy in a type 2 diabetes rat model. *PLoS One.* (2014) 9:e104771. 10.1371/journal.pone.0104771 25136835 PMC4138036

[125] Qiu Z He Y Ming H Lei S Leng Y Xia ZY. Lipopolysaccharide (LPS) Aggravates High Glucose- and Hypoxia/Reoxygenation-Induced Injury through Activating ROS-Dependent NLRP3 Inflammasome-Mediated Pyroptosis in H9c2 Cardiomyocytes. *J Diabetes Res.* (2019) 2019:8151836. 10.1155/2019/8151836 30911553 PMC6398034

[126] Yang F Li A Qin Y Che H Wang Y Lv J A novel circular RNA mediates pyroptosis of diabetic cardiomyopathy by functioning as a competing endogenous RNA. *Mol Ther Nucleic Acids.* (2019) 17:636–43. 10.1016/j.omtn.2019.06.026 31400606 PMC6700436

[127] Ibáñez B Heusch G Ovize M Van de Werf F. Evolving therapies for myocardial ischemia/reperfusion injury. *J Am Coll Cardiol.* (2015) 65:1454–71. 10.1016/j.jacc.2015.02.032 25857912

[128] Takahashi M. Role of NLRP3 inflammasome in cardiac inflammation and remodeling after myocardial infarction. *Biol Pharm Bull.* (2019) 42:518–23. 10.1248/bpb.b18-00369 30930410

[129] Ding S Liu D Wang L Wang G Zhu Y. Inhibiting microrna-29a protects myocardial ischemia-reperfusion injury by targeting SIRT1 and suppressing oxidative stress and NLRP3-mediated pyroptosis pathway. *J Pharmacol Exp Ther.* (2020) 372:128–35. 10.1124/jpet.119.256982 31481517

[130] Deftereos S Giannopoulos G Angelidis C Alexopoulos N Filippatos G Papoutsidakis N Anti-inflammatory treatment with colchicine in acute myocardial infarction: a pilot study. *Circulation.* (2015) 132:1395–403. 10.1161/circulationaha.115.017611 26265659

[131] Pomerantz BJ Reznikov LL Harken AH Dinarello CA. Inhibition of caspase 1 reduces human myocardial ischemic dysfunction via inhibition of Il-18 and Il-1beta. *Proc Natl Acad Sci USA.* (2001) 98:2871–6. 10.1073/pnas.041611398 11226333 PMC30232

[132] Kawaguchi M Takahashi M Hata T Kashima Y Usui F Morimoto H Inflammasome activation of cardiac fibroblasts is essential for myocardial ischemia/reperfusion injury. *Circulation.* (2011) 123:594–604. 10.1161/circulationaha.110.982777 21282498

[133] Syed FM Hahn HS Odley A Guo Y Vallejo JG Lynch RA Proapoptotic effects of caspase-1/interleukin-converting enzyme dominate in myocardial ischemia. *Circ Res.* (2005) 96:1103–9. 10.1161/01.RES.0000166925.45995.ed 15845887

[134] Koshinuma S Miyamae M Kaneda K Kotani J Figueredo VM. Combination of necroptosis and apoptosis inhibition enhances cardioprotection against myocardial ischemia-reperfusion injury. *J Anesth.* (2014) 28:235–41. 10.1007/s00540-013-1716-3 24113863

[135] Mocanu MM Baxter GF Yellon DM. Caspase inhibition and limitation of myocardial infarct size: protection against lethal reperfusion injury. *Br J Pharmacol.* (2000) 130:197–200. 10.1038/sj.bjp.0703336 10807653 PMC1572087

[136] Do Carmo H Arjun S Petrucci O Yellon DM Davidson SM. the caspase 1 inhibitor VX-765 protects the isolated rat heart via the risk pathway. *Cardiovasc Drugs Ther.* (2018) 32:165–8. 10.1007/s10557-018-6781-2 29582211 PMC5958154

[137] Audia JP Yang XM Crockett ES Housley N Haq EU O’Donnell K Caspase-1 inhibition by VX-765 administered at reperfusion in P2Y(12) receptor antagonist-treated rats provides long-term reduction in myocardial infarct size and preservation of ventricular function. *Basic Res Cardiol.* (2018) 113:32. 10.1007/s00395-018-0692-z 29992382 PMC6396295

[138] Korantzopoulos P Letsas KP Tse G Fragakis N Goudis CA Liu T. Inflammation and atrial fibrillation: a comprehensive review. *J Arrhythm.* (2018) 34:394–401. 10.1002/joa3.12077 30167010 PMC6111477

[139] Yao C Veleva T Scott L Jr. Cao S Li L Chen G Enhanced cardiomyocyte NLRP3 inflammasome signaling promotes atrial fibrillation. *Circulation.* (2018) 138:2227–42. 10.1161/circulationaha.118.035202 29802206 PMC6252285

[140] Chen G Chelu MG Dobrev D Li N. Cardiomyocyte inflammasome signaling in cardiomyopathies and atrial fibrillation: mechanisms and potential therapeutic implications. *Front Physiol.* (2018) 9:1115. 10.3389/fphys.2018.01115 30150941 PMC6100656

[141] Colston JT Boylston WH Feldman MD Jenkinson CP de la Rosa SD Barton A Interleukin-18 Knockout Mice Display Maladaptive Cardiac Hypertrophy in Response to Pressure Overload. *Biochem Biophys Res Commun.* (2007) 354:552–8. 10.1016/j.bbrc.2007.01.030 17250807 PMC1847636

[142] Li R Lu K Wang Y Chen M Zhang F Shen H Triptolide attenuates pressure overload-induced myocardial remodeling in mice via the inhibition of NLRP3 inflammasome expression. *Biochem Biophys Res Commun.* (2017) 485:69–75. 10.1016/j.bbrc.2017.02.021 28202417

[143] Wang Y Wu Y Chen J Zhao S Li H. Pirfenidone attenuates cardiac fibrosis in a mouse model of TAC-induced left ventricular remodeling by suppressing NLRP3 inflammasome formation. *Cardiology.* (2013) 126:1–11. 10.1159/000351179 23839341

[144] Wang F Liang Q Ma Y Sun M Li T Lin L silica nanoparticles induce pyroptosis and cardiac hypertrophy via ROS/NLRP3/Caspase-1 pathway. *Free Radic Biol Med.* (2022) 182:171–81. 10.1016/j.freeradbiomed.2022.02.027 35219847

[145] Yue R Zheng Z Luo Y Wang X Lv M Qin D NLRP3-mediated pyroptosis aggravates pressure overload-induced cardiac hypertrophy, fibrosis, and dysfunction in mice: cardioprotective role of irisin. *Cell Death Discov.* (2021) 7:50. 10.1038/s41420-021-00434-y 33723236 PMC7961005

